# Impact of discrimination on training and career of radiation oncologists in France

**DOI:** 10.1016/j.ctro.2024.100840

**Published:** 2024-08-13

**Authors:** Sabrina Aziez, Cécile Evin, David Azria, Erik Montpetit, Youssef Gannam, Yasmine El Houat, Amandine Ruffier, Véronique Vendrely, Anne Laprie, Florence Huguet

**Affiliations:** a Department of Radiation Oncology, Tenon Hospital, AP-HP, Sorbonne University, Paris, France; b Department of Radiation Oncology, Institut du Cancer de Montpellier, Montpellier, France; c Department of Radiation Oncology, Hôpital Privé Océane, Vannes, France; d Department of Radiation Oncology, Institut de Cancérologie de l’Ouest, Angers, France; e Department of Radiation Oncology, Centre Hospitalier Universitaire Vaudois, Lausanne, Suisse; f Department of Radiation Oncology, Clinique Victor Hugo, Le Mans, France; g Department of Radiation Oncology, Centre Hospitalier Universitaire Bordeaux, Bordeaux, France; h Department of Radiation Oncology, Institut Universitaire du Cancer de Toulouse, Toulouse, France

**Keywords:** Career, Discrimination, Gender equity, Leadership, Oncology, Radiation therapy, radiotherapy, pay gap, Survey

## Abstract

**Purpose:**

In France, radiation oncologists are predominantly men with only 44 % of women. Many studies have highlighted gender disparities in medicine. The main objective of our study was to assess the impact of discriminations on radiation oncologists’ career.

**Materials and methods:**

An anonymous online questionnaire, adapted from the one used by the ESMO W4O group, was sent to all radiation oncologists in France between March and June 2022. It included questions related to professional experience, gender, socio-ethnicity, sexual orientation, and personal life.

**Results:**

Among the 999 radiation oncologists and 168 residents in France, 225 questionnaires were collected (19.2 %). Among the respondents, 60 % were women and 25 % were residents. The mean age was 39.2 years (range: 25–78). The career satisfaction rate was 92 %, with no gender difference. Gender was considered to have a negative impact on the career development by 65 % of women. Social origin was an obstacle to career development for 37 % of all the respondents, and ethnic origin for 25 %. Sixty two percent of women reported having experienced inappropriate behavior or sexual harassment in their workplace, 38 % felt that having a child had “extremely“ or ”very“ much impacted their career versus 8.5 % of men (p < 0.001). The most popular proposals for improvement were the creation of a network of women radiation oncologists with specific educational programs and the addition of quotas in institutions and key positions.

**Conclusions:**

This study is the first one assessing the various type of discrimination experienced by radiation oncologists in France. We make a few proposals for improvement of training and working conditions, regardless of the origin and gender.

## Highlights

• First assessing the various types of discrimination experienced by women radiation oncologists.
• Gender was considered to have a negative impact on the career development of women radiation oncologists.
• Two-thirds of women radiation oncologists experienced inappropriate behavior in their workplace.
• Social and ethnic origin were considered obstacles to career development.
• Proposals are made for improvement of training and working conditions.

## Introduction

Women represent 75 % of the health workforce but 52 % of medical personnel in France [1], [2]. However, while there are currently more women than men in medical schools, women are still underrepresented in the senior hierarchical functions of French hospitals [1], [3]. Despite social advances since the 2000s, women are generally paid less than their male colleagues [4], [5], [6], [7]. Furthermore, in academia, women represent 30 % of the authors of scientific articles, 33 % of the first authors, but only 18 % of the last authors and they are less published in highly cited scientific journals compared to men and less cited than men [8], [9], [10]. Moreover, doctors and medical students from lesbian, gay, bisexual, transgender and queer, intersex (LGBTQI) communities reported being the target of discrimination and being victims of ostracism in medicine [11], [12], [13], [14], [15], [16].

In oncology, the issue of diversity has been the subject of several publications. In 2018, ESMO published a study on the role of women and people from minorities working in positions of responsibility in scientific oncologic societies [17], [18]. To better assess the impact of gender discrimination, ESMO has developed a dedicated committee called Women for Oncology (W4O) [19]. In the same way as the W4O group, several working groups have published their data on the quality of life and discrimination among oncologists in Europe and internationally [20], [21], [22], [23], [24]. In radiation oncology, significant discrimination against physicians from minorities has been reported [11], [12], [13], [14], [15], [16]. While diversity is a very broad and rich research theme in Anglo-Saxon countries and in the rest of the world, it remains a subject that is not frequently addressed in France, including within radiation oncology. In 2021, there were 999 radiation oncologists (RO) in France, including 435 women, a proportion that has increased significantly, from 39 % in 2012 to reach 43.4 % today [25]. We conducted this study to assess the situation among French RO, and then suggest ideas for improvement.

## Methods

We used an online anonymous questionnaire, adapted from the one used in 2021 by the ESMO W4O group [19], [26], [27]. After obtaining the authorization from W4O group to use it, we translated into French and we added questions about social origin. It contained 76 questions separated in five sections related to demographics and professional environment, experience and career, personal life, as well as a section dedicated to academic research. In addition to gender bias, we extended our questions to other type of discrimination such as racial, social, religious, and sexual orientation bias. In France, registering data about ethnicity, religion or sexual orientation is not permitted so we used indirect questions to try to ascertain participants views relating to these factors. This study was registered with the French national committee for information and freedom (Commission Nationale de l’Information et des Libertés, CNIL) in March 2022. The questionnaire was piloted on five volunteers. Then, it was sent to all the residents and ROs in France in March 2022 with the help of our leading radiation oncology societies, such as the French society of RO (SFRO), the association of RO’s residents (SFJRO), and other organisations. Results are presented overall and by respondent’s gender. We used Fisher’s exact test, Mann-Whitney *U* test and the Chi-squared test to examine for corresponding associations setting a significance level of α = 5 %.’ .

## Results

### Population and demographics (Table 1)

Between March and June 2022, among the 999 radiation oncology practitioners and 168 residents in France, 232 questionnaires were collected and 225 analyzed (19.2 %). Seven were excluded because not returned by’from ROs. Among those 225 respondents, 135 (60 %) were women and 90 were men (40 %). The mean age was 39.2 years (range: 25–78). Women were younger (37.7 years versus 41.6 years for men, p = 0.019). One hundred sixty-eight respondents (75 %) were fully qualified practitioners and 57 (25 %) were residents. Among the practitioners, 42 % were practicing in a cancer center, 25 % in a university hospital, 23 % in a private institution and 10 % in another type of service. More men worked in private institution (43 % versus 23 %, p < 0.01). Respondents were mainly born in France (86 %); thirty-two (14 %) were born abroad, in North Africa (n = 12), Europe (n = 8), or Sub-Saharan Africa (n = 6), or elsewhere (n = 6).

**Table 1:** Characteristics of the population.

	All (n = 225)	Men (n = 90)	Women (n = 135)	p
**Age (mean)**	**39.2**	**41.6**	**37.7**	**0.02**

Country of birth				
France	193 (86 %)	82 (91 %)	111 (82 %)	0.06
Other	32 (14 %)	8 (9 %)	24 (18 %)	

**Do you live alone?**				
**Yes**	**54 (24 %)**	**15 (17 %)**	**39 (29 %)**	**0.035**
**No**	**171 (76 %)**	**75 (83 %)**	**96 (71 %)**	

Marital status				
Single	32 (14 %)	10 (11 %)	22 (16 %)	0.16
Couple	61 (27 %)	27 (31 %)	34 (25 %)	
Married	122 (54 %)	51 (57 %)	71 (53 %)	
Divorced	9 (4 %)	1 (1 %)	8 (6 %)	
Widow	1 (1 %)	1 (1 %)	0 (0 %)	

Parent				
Yes	133 (59 %)	59 (66 %)	74 (55 %)	0.11
No	92 (41 %)	31 (34 %)	61 (45 %)	

**Number of children**	**2.29**	**2.56**	**2.07**	**<0.01**

Facility				
University hospital	58 (26 %)	21 (23 %)	37 (27 %)	0.2
General hospital	15 (6.7 %)	4 (4.4 %)	11 (8 %)	
Private clinic	52 (23 %)	28 (31 %)	24 (18 %)	
Cancer center	95 (42 %)	35 (39 %)	60 (44 %)	
Other	5 (2.3 %)	2 (2.6 %)	3 (2 %)	

One hundred twenty-two (54 %) were married, and 133 (59 %) had children. Men had significantly more children than women (2.6 versus 2.1 p < 0.01) and women tended to live alone more often than men (29 % versus 17 % p = 0.035).

### Professional experience and career

Overall, the career satisfaction rate was 92 %, with no gender difference for qualified ROs and residents. We did not find any difference in the number of working hours between men and women, except for the time dedicated to management, which was higher for men (10 % of time dedicated to management for women and 15 % for men p = 0,046). Half of the respondents (52 %) gave a high importance to progression in their careers, independent of gender.

**Fig. 1:**
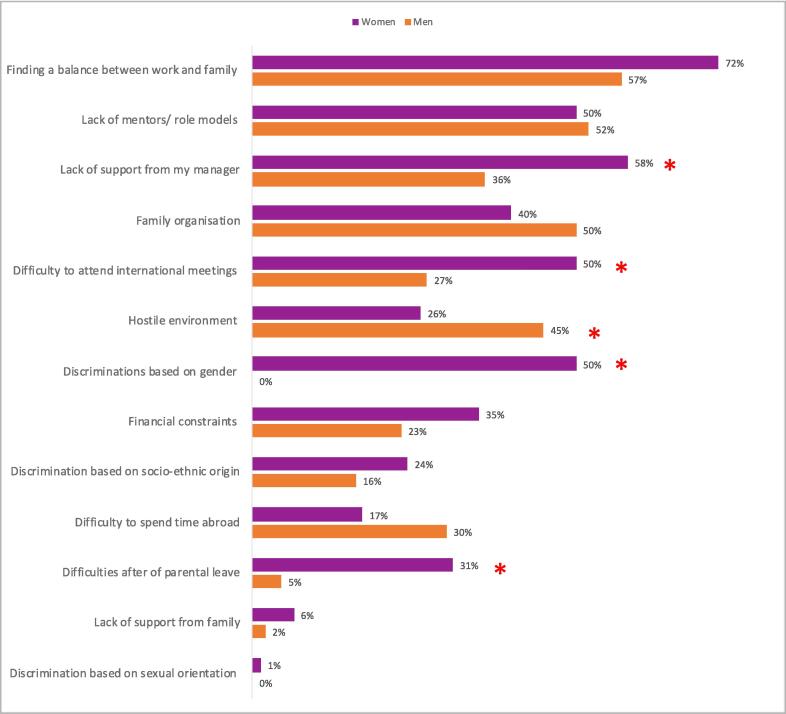
Most important obstacles in the career of radiation oncologists (* means the difference is statistically significant).

For all respondents, the main obstacles reported during their careers were finding a balance between work and family (66 %), the lack of mentors/role models (51 %), and the lack of support from their manager (50 %). Women reported significantly more often that they lacked support from their managers compared to men (58 % versus 36 % respectively, p = 0.024). Women also had higher rates of difficulty returning to work after parental leave (31 % versus 4.5 %, p < 0.001) and more women experienced barriers to international meeting attendance (58 % versus 36 %, p = 0.024) (Fig. 1). When asked if their gender had a negative impact on their career, 65 % of women reported a negative impact versus 12.7 % of men (p < 0.001). Forty-four percent of women had experienced discrimination from patients because of their gender.

**Fig. 2:**
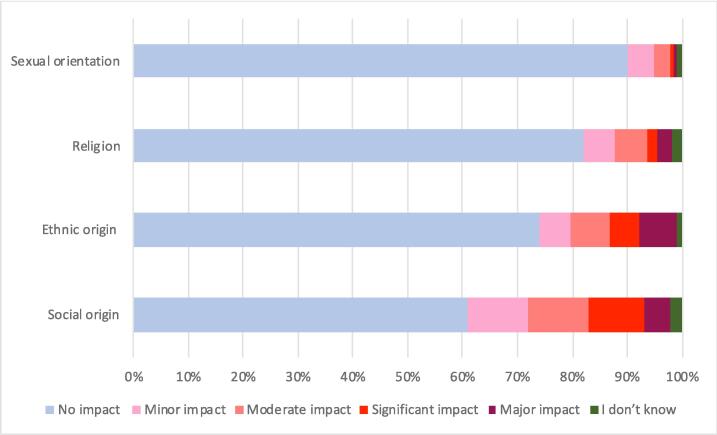
Individual factors impacting negatively the career of radiation oncologists.

Twenty-five percent of respondents considered that their ethnicity had a negative impact on their career development, 37 % considered that they were negatively impacted by their social background, 16 % by their religion, and 9 % by their sexual orientation (Fig. 2). Conversely, 60 % of respondents declared having more opportunities because of their social background. RO not born in France reported significantly more negative impact of their ethnicity compared to those born in France (72 % versus 18 %, p < 0.001), their religion (35 % versus 13 %, p < 0.01), and their social background (66 % versus 32 %, p < 0.001). Twenty percent of all respondents experienced discrimination from patients because of their ethnicity compared to 53 % for the cohort not born in France. ROs experiencing discrimination from patients had lower professional satisfaction rates than those who did not (8.7 % versus 91 %, p < 0.001).

### Women in the workplace

**Fig. 3:**
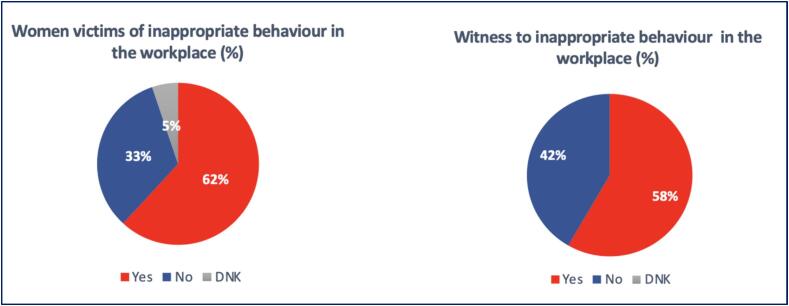
Summary of responses as victims of and witness to inappropriate behaviour/sexual assault.

In the workplace, 84 women (62 %) experienced inappropriate behavior or another type of harassment compared to 7 % of men (p < 0.001) (Fig. 3). Most of the time, it consisted of sexist remarks (58 %) but 30 % of women experienced inappropriate sexual advances, and 4 % had been sexually assaulted in their workplace. At the same time, 56 % of all respondents had witnessed inappropriate behavior or another type of harassment, with a difference between genders (67 % of women versus 41 % of men, p < 0.001) (Fig. 3).

Only 39 respondents (17 %) had reported harassment to a superior, more frequently women than men (22 % versus 10 %, p < 0.001). The reasons for not reporting were mainly fear of reprisal (74 %) and lack of importance (44 %) This last reason was more often chosen by men (66 % versus 37 %, p < 0.001). Seven percent of respondents acknowledged having had discriminatory behavior towards patients and 7 % towards colleagues. Among the respondents acknowledging discriminatory behavior, residents were overrepresented (14 % versus 4.2 %, p = 0.03).

### Personal life

Thirty-eight percent of women felt that having a child had “extremely“ or ”very“ much negatively impacted their career compared to 8.5 % of men (p < 0.001) and 23.5 % believed that “taking parental leave” had “extremely” or “very” impacted their career compared to 8.5 % of men (p = 0.03). For men, their working hours and careers had significantly impacted more on their time with their children compared to women (46 % versus 38 %, p = 0.014).

Among people living as a couple, the distribution of household tasks, preparation of meals, childcare, and laundry were significantly more often considered the responsibility of women. A greater proportion of men delegated −often or always- these tasks to women as opposed to the other way around (21 % versus 5.3 %, p < 0.01).

### Suggestions for improvement

**Fig. 4:**
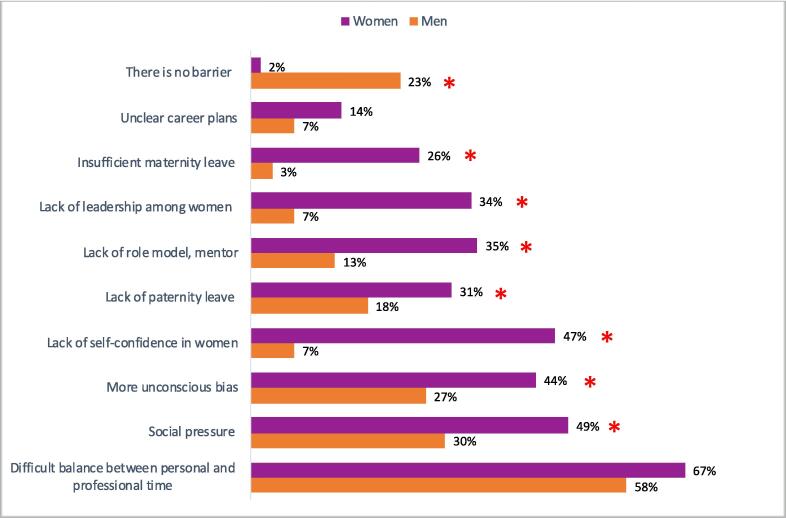
Barriers to gender equality in radiation oncology in France according to gender of respondent. (* means the difference is statistically significant).

Identified barriers to gender equality reported by the respondents are presented in Fig. 4. Interestingly, 23 % of men reported no barriers to gender parity, compared to only 1.5 % of women (p < 0.001). Concerning the perception of evolution towards gender parity, men tended to perceive more major progress (9 %) in closing gender gap than women (0.7 %), and, similarly, 10 % of women felt that there had been no change in gender equality compared to 1 % of men (p < 0.001).

**Fig. 5:**
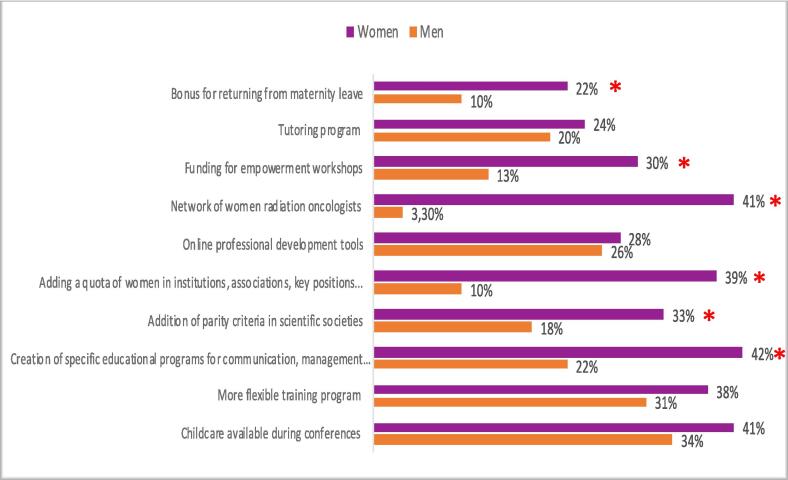
Responses to suggested proposals to support equality and diversity in radiation oncology in France’. (* means the difference is statistically significant).

The most popular proposals for improvement in gender parity in radiation oncology were the “Creation of specific educational programs” (42 % of women versus 22 % of men, p < 0.01), the creation of a “Network of women ROs” (41 % versus 3.3 %; p < 0.001), and the “Addition of quotas in institutions, associations, and key positions” (39 % versus 10 %; p < 0.001) (Fig. 5). We can also keep in mind the promotion of « Family friendly facilities at oncology events” desired by 39 % of the respondents with no difference regarding gender of respondents.

## Discussion

This study is the first to our knowledge in France to evaluate the impact of various types of discrimination related to gender, socio-ethnic origin, religion, and sexual orientation on training and career of ROs. We collected 225 questionnaires representing a response rate of 19 % over just two months.

In our study, 65 % of responding women reported that gender had a negative impact on their career compared to 12.7 % of men (p < 0.001). We also reported than 62 % of female respondents were victims of inappropriate behavior or sexual harassment in the workplace. These data suggested a strong tendency to ignore these negative behaviours as more than half of the respondents have witnessed an inappropriate behavior and only 17 % have reported this type of action. Regarding other forms of discrimination, one person in four considered their ethnicity as a negative factor in their career and one person in three their social background, a trend significantly higher among people not born in France. This discrimination has a real impact on the quality of life at work since people who have suffered discrimination linked to ethnic origin have a much lower job satisfaction rate, proving once again that discrimination matters.

Most of these results are consistent with those reported very recently in the last European study performed by the W4O group. In their study, the proportion of respondents declaring that gender had a major or significant impact on their professional career was higher in women than in men (60.4 % versus 19.1 %, p < 0.0001) [26]. We observed a higher number of episodes of inappropriate behavior than in other surveys of this kind, in the literature ranging from 28.8 to 52 % [14], [15], [17], [32]. In our study, and in contrast to the recent study by Linardou et al, ethnic and social origin had a negative impact on the careers of ROs [26].

Some of the gender disparities observed in our study could be explained by, on one hand, a greater negative impact on women's careers of parenthood and taking leave and on the other, by the fact that for equivalent working time between men and women, the distribution of household tasks remains unbalanced impinging on the conduct of other projects. For men, work and career significantly affected their time devoted to children (significant impact: 46 % versus 38 %, p = 0.014), that women cannot make as much concessions on time spent with children than their male colleagues. Indeed, in the study conducted among American ROs published by Holliday et al, 70 % of men tended to leave child-related tasks to their partner, unlike 35 % of women [28]. Furthermore, women reported less support from their superiors, social pressure, and the persistence of unconscious bias. We observed also a difference in the perception of changes in terms of gender inequalities, with men more often reporting progress than women. As in the 2018 ESMO W4O study, 39 % of men felt there had been significant or important changes compared to only 14 % of women [19].

To our knowledge, this study is the first in France to assess discrimination among medical doctors, with the support and enthusiasm of France's leading radiation oncology societies. Although our sample consisted of 60 % women and rather young (average age 39), it included more men than other studies, as well as more private practitioners, making the results more representative.

One of the main limitations of our study is a relatively low participation rate of 19 %, however comparable to that of the Spanish study of 14.7 % (318 responses/2151 oncologists) [22]. There was an overrepresentation of women compared to the proportion of women in radiation oncology (60 % versus 44 %) but closer to reality than other studies. One of our main biases was the imprecise definition of “inappropriate behavior and situations of sexual harassment” because we did not anticipate such a high incidence (62 % of female victims) and because we used the same terms as the W40 2021 survey to be able to compare our results.

Another limitation of this study was that it did not assess the signs of burnout and psychological disorders which can be accentuated in the event of discrimination [29]. In our assessment, we did not take into account other types of discrimination, particularly related to physical appearance, disabilities, and age, which are rarely assessed in studies. Finally, the major limitation of our study was the impossibility to ask the participants about their ethnic origin, religion, and sexual orientation to comply with of French legislation. We had to study discrimination related to these questions indirectly.

The most popular proposals for improvement in gender parity in radiation oncology were the “Creation of specific educational programs”, the creation of a “Network of women ROs”, and the ”Addition of quotas in institutions, associations and key positions“. Proposals commonly found in the literature are the establishment of training programs dedicated to women [22], [40] or support dedicated to the career of women ROs [40], [41], [42], [43], [44].: Less common solutions discussed were improvement of working conditions (including more flexibility), childcare at congresses [22], [45] and encouragement for fathers to take paternity leave [46]. Another major step would be the establishment of a formal mentoring system, key to many recommendations from scientific societies [40], [41], [47], [48], [49]. In addition, various studies insist on the importance of making regular evaluations. It would be useful to develop systems for measuring and monitoring progress on inclusion and diversity in the workplace [41], [48].

In addition to the ethical aspect of promoting equality, diversity amongst health professional staff must be promoted as a proven asset. Several studies have shown the positive impact of having women and people from minority backgrounds train as doctors in relation to the doctor-patient relationship [30], [31], the distribution of care provision [32], [33], [34], and the quality of care [35], [36], [37], [38], [39]. The greater difference in awareness of discrimination against patients among residents than among practitioners is a sign of improved working conditions and hope for the future. Beginning by recognizing the limits of our practice and our training is an essential first step, even if other studies are necessary to better assess the impact of discrimination and to follow future improvements. We hope that other specialties in France, especially medical oncology, will in turn assess the impact of discrimination in their professional context.

## Conclusions

This study provides an initial assessment of the different forms of discrimination experienced by ROs in France during their training and practice. It reports on the main obstacles encountered in their careers. We show just how far we still have to go to achieve gender equality. For the first time in France, we have carried out an analysis of other types of discrimination experienced in the workplace. In addition, this study has enabled us to identify a few important factors to help in understanding these inequalities, and to create the first inclusion committee in the French Radiotherapy Oncology Society (SFRO). Finally, we propose a few suggestions for improvement in future years, to maintain vigilance over disparities in our specialty and thus guarantee the best training and working conditions for radiation oncologists, whatever their origins and gender. These measures for greater equality within our specialty are not only ethical, but bring value to patients and health professionals alike.’

## CRediT authorship contribution statement

**Sabrina Aziez:** Conceptualization, Investigation, Formal analysis, Writing – original draft. **Cécile Evin:** Writing – review & editing. **David Azria:** Resources. **Erik Montpetit:** Resources. **Youssef Gannam:** Resources. **Yasmine El Houat:** Writing – review & editing. **Amandine Ruffier:** Writing – review & editing, Validation. **Véronique Vendrely:** Writing – review & editing, Validation. **Anne Laprie:** Writing – review & editing, Validation. **Florence Huguet:** Conceptualization, Methodology, Writing – review & editing, Supervision.

## Declaration of competing interest

The authors declare that they have no known competing financial interests or personal relationships that could have appeared to influence the work reported in this paper.

## References

[1] Journée internationale des droits des femmes - L’AP-HP s’engage en faveur de l’égalité entre les femmes et les hommes [Internet]. [cité 22 mai 2022]. Disponible sur: https://www.aphp.fr/contenu/journee-internationale-des-droits-des-femmes-lap-hp-sengage-en-faveur-de-legalite-entre-les.

[2] Rosso C., Léger A., Steichen O. (2019). Plafond de verre pour les femmes dans les carrières hospitalo-universitaires en France. Rev Médecine Interne Févr.

[3] CNG -Résultats parité HU-2021-Synthèse.pdf [Internet]. [cité 22 mai 2022]. Disponible sur: https://www.cng.sante.fr/sites/default/files/media/2022-03/2021_HU_Synth%C3%A8se.pdf.

[4] Boniol M, McIsaac M, Xu L, Wuliji T, Diallo K, Campbell J. Gender equity in the health workforce: Analysis of 104 countries. 8.

[5] Laine C., Turner B.J. (2004). Unequal pay for equal work: the gender gap in academic medicine. Ann Intern Med.

[6] Wright A.L., Schwindt L.A., Bassford T.L., Reyna V.F., Shisslak C.M., St Germain P.A. (2003). Gender differences in academic advancement: patterns, causes, and potential solutions in one US College of Medicine. Acad Med J Assoc Am Med Coll.

[7] Guss Z.D., Chen Q., Hu C., Guss L.G., DeWeese T.L., Terezakis S.A. (2019). Differences in physician compensation between men and women at United States Public Academic Radiation Oncology Departments. Int J Radiat Oncol Biol Phys.

[8] Bendels M.H.K., Müller R., Brueggmann D., Groneberg D.A. (2018). Gender disparities in high-quality research revealed by Nature Index journals. PLoS One.

[9] Elsevier. Elsevier Connect. [cité 24 mai 2022]. Elsevier’s reports on gender in research. Disponible sur: https://www.elsevier.com/connect/gender-report.

[10] Zayed S., Qu X.M., Warner A., Zhang T.W., Laba J.M., Rodrigues G.B. (2020). Are female radiation oncologists still underrepresented in the published literature? An analysis of authorship trends during the past decade. Adv Radiat Oncol Juin.

[11] Suneja G, Mattes MD, Mailhot Vega RB, Escorcia FE, Lawton C, Greenberger J, et al. Pathways for Recruiting and Retaining Women and Underrepresented Minority Clinicians and Physician Scientists Into the Radiation Oncology Workforce: A Summary of the 2019 ASTRO/NCI Diversity Symposium Session at the ASTRO Annual Meeting. Adv Radiat Oncol. 2020;5(5):798‑803. 10.1016/j.adro.2020.05.003 PMC7557133 33083641

[12] Nead K.T., Linos E., Vapiwala N. (2019). Increasing diversity in radiation oncology: a call to action. Adv Radiat Oncol Juin.

[13] Deville C., Chapman C.H., Burgos R., Hwang W.T., Both S., Thomas C.R. (2014). Diversity by race, Hispanic ethnicity, and sex of the United States medical oncology physician workforce over the past quarter century. J Oncol Pract Sept.

[14] Feral-Pierssens A.L., Avondo A., Apard M., Monguillet J., Gonot A., De Stefano C. (2021). Parité de genre dans les publications scientifiques françaises : le plafond de verre. L’encéphale.

[15] Ginther D.K., Haak L.L., Schaffer W.T., Kington R. (2012). Are race, ethnicity, and medical school affiliation associated with NIH R01 type award probability for physician investigators?. Acad Med J Assoc Am Med Coll.

[16] Check H.E. (2015). Racial bias continues to haunt NIH grants. Nature.

[17] Hofstädter-Thalmann E., Dafni U., Allen T., Arnold D., Banerjee S., Curigliano G. (2018). Report on the status of women occupying leadership roles in oncology. ESMO Open.

[18] Chapman C.H., Jagsi R. (2017). The ethical imperative and evidence-based strategies to ensure equity and diversity in radiation oncology. Int J Radiat Oncol.

[19] Banerjee S, Dafni U, Allen T, Arnold D, Curigliano G, Garralda E, et al. Gender-related challenges facing oncologists: the results of the ESMO Women for Oncology Committee survey. ESMO Open [Internet]. 2018 [cité 9 janv 2022];3(6). Disponible sur: https://www.esmoopen.com/article/S2059-7029(20)30217-9/fulltext. 10.1136/esmoopen-2018-000422 PMC6157518 30273420

[20] Dieci MV, Massari F, Giusti R, Inno A, Lombardi G, Noto L, et al. Gender influence on professional satisfaction and gender issue perception among young oncologists. A survey of the Young Oncologists Working Group of the Italian Association of Medical Oncology (AIOM). ESMO Open [Internet]. 2018 [cité 9 janv 2022];3(6). Disponible sur: https://www.esmoopen.com/article/S2059-7029(20)30210-6/fulltext#supplementaryMaterial. 10.1136/esmoopen-2018-000389 PMC6212682 30425842

[21] Salem R., Haibe Y., Dagher C., Salem C., Shamseddine A., Bitar N. (2019). Female oncologists in the Middle East and North Africa: progress towards gender equality. ESMO Open.

[22] Elez E., Ayala F., Felip E., García Campelo R., García Carbonero R., García Donás J. (2021). Gender influence on work satisfaction and leadership for medical oncologists: a survey of the Spanish Society of Medical Oncology (SEOM). ESMO Open.

[23] Bajpai J., Mailankody S., Nair R., Surappa T.S., Gupta S., Prabhash K. (2020). Gender climate in Indian oncology: national survey report. ESMO Open Avr.

[24] Garrido P., Adjei A.A., Bajpai J., Banerjee S., Berghoff A.S., Choo S.P. (2021). Has COVID-19 had a greater impact on female than male oncologists? Results of the ESMO Women for Oncology (W4O) Survey. ESMO Open Juin.

[25] Masson E. EM-Consulte. [cité 11 janv 2022]. Démographie des internes en oncologie–radiothérapie en France en 2008 : état et perspectives pour les trois prochaines années. Disponible sur: https://www.em-consulte.com/article/214319/demographie-des-internes-en-oncologienradiotherapi. 10.1016/j.canrad.2009.01.005 19268618

[26] Linardou H., Adjei A.A., Bajpai J., Banerjee S., Berghoff A.S., Mathias C.C. (2023). Challenges in oncology career: are we closing the gender gap? Results of the new ESMO Women for Oncology Committee survey. ESMO Open Avr.

[27] ESMO. Survey on Challenges Facing Oncology Professionals [Internet]. [cité 21 août 2022]. Disponible sur: https://www.esmo.org/career-development/women-for-oncology/esmo-w4o-observatory/survey-on-challenges-facing-oncology-professionals.

[28] Holliday E.B., Ahmed A.A., Jagsi R., Stentz N.C., Woodward W.A., Fuller C.D. (2015). Pregnancy and Parenthood in Radiation Oncology, Views and Experiences Survey (PROVES): Results of a Blinded Prospective Trainee Parenting and Career Development Assessment. Int J Radiat Oncol Biol Phys.

[29] Sojo V.E., Wood R.E., Genat A.E. (2016). Harmful workplace experiences and women’s occupational well-being: a meta-analysis. Psychol Women q.

[30] Cooper-Patrick L., Gallo J.J., Gonzales J.J., Vu H.T., Powe N.R., Nelson C. (1999). Race, gender, and partnership in the patient-physician relationship. JAMA.

[31] Disparities in Patient Experiences, Health Care Processes, and Outcomes: The Role of Patient-Provider Racial, Ethnic, and Language Concordance | Commonwealth Fund [Internet]. [cité 28 janv 2022]. Disponible sur: https://www.commonwealthfund.org/publications/fund-reports/2004/jul/disparities-patient-experiences-health-care-processes-and.

[32] Kumar D., Schlundt D.G., Wallston K.A. (2009). Patient-physician race concordance and its relationship to perceived health outcomes. Ethn Dis.

[33] Marrast LM, Zallman L, Woolhandler S, Bor DH, McCormick D. Minority physicians’ role in the care of underserved patients: diversifying the physician workforce may be key in addressing health disparities. JAMA Intern Med. 2014;174(2):289‑91. 10.1001/jamainternmed.2013.12756 24378807

[34] Moy E, Bartman BA. Physician race and care of minority and medically indigent patients. JAMA. 1995;273(19):1515‑20. 7739078

[35] Greenwood BN, Hardeman RR, Huang L, Sojourner A. Physician–patient racial concordance and disparities in birthing mortality for newborns. Proc Natl Acad Sci. 2020;117(35):21194‑200. 10.1073/pnas.1913405117 PMC7474610 32817561

[36] Tsugawa Y, Jena AB, Figueroa JF, Orav EJ, Blumenthal DM, Jha AK. Comparison of hospital mortality and readmission rates for medicare patients treated by male vs female physicians. JAMA Intern Med. 2017;177(2):206‑13. 10.1001/jamainternmed.2016.7875 PMC5558155 27992617

[37] Wallis CJ, Ravi B, Coburn N, Nam RK, Detsky AS, Satkunasivam R. Comparison of postoperative outcomes among patients treated by male and female surgeons: a population based matched cohort study. BMJ. 2017;359:j4366. 10.1136/bmj.j4366 PMC6284261 29018008

[38] Greenwood BN, Carnahan S, Huang L. Patient-physician gender concordance and increased mortality among female heart attack patients. Proc Natl Acad Sci U S A. 2018;115(34):8569‑74. 10.1073/pnas.1800097115 PMC6112736 30082406

[39] Roter DL, Hall JA, Aoki Y. Physician gender effects in medical communication: a meta-analytic review. JAMA. 2002;288(6):756‑64. 10.1001/jama.288.6.756 12169083

[40] Lightfoote J.B., Fielding J.R., Deville C., Gunderman R.B., Morgan G.N., Pandharipande P.V. (2014). Improving diversity, inclusion, and representation in radiology and radiation oncology part 1: why these matter. J Am Coll Radiol JACR Juill.

[41] Suneja G., Mattes M.D., Mailhot Vega R.B., Escorcia F.E., Lawton C., Greenberger J. (2020). Pathways for recruiting and retaining women and underrepresented minority clinicians and physician scientists into the Radiation Oncology Workforce: A summary of the 2019 ASTRO/NCI Diversity Symposium Session at the ASTRO Annual Meeting. Adv Radiat Oncol.

[42] Odei B., Kahn J., Holliday E.B., Diaz D.A., Bello-Pardo E., Odei J. (Oct 2021). Where are the women in radiation oncology? A cross-sectional multi-specialty comparative analysis. Adv Radiat Oncol.

[43] Lin M.P., Lall M.D., Samuels-Kalow M., Das D., Linden J.A., Perman S. (2019). Impact of a women-focused professional organization on academic retention and advancement: perceptions from a qualitative study. Acad Emerg Med off J Soc Acad Emerg Med Mars.

[44] Mansh M., Garcia G., Lunn M.R. (2015). From patients to providers: changing the culture in medicine toward sexual and gender minorities. Acad Med J Assoc Am Med Coll Mai.

[45] Robles J., Anim T., Wusu M.H., Foster K.E., Parra Y., Amaechi O. (2021). An approach to faculty development for underrepresented minorities in medicine. South Med J Sept.

[46] Siddiqui OM, Savla B, Chowdhary M, McAvoy SA, Mishra MV. From beaming cancer to beaming parent: paternity leave experiences in radiation oncology. Int J Radiat Oncol Biol Phys. 2021;111(3):e339‑40. 10.1016/j.ijrobp.2022.04.031 35500797

[47] Deanna R, Merkle BG, Chun KP, Navarro-Rosenblatt D, Baxter I, Oleas N, et al. Community voices: the importance of diverse networks in academic mentoring. Nat Commun. 2022;13(1):1681. 10.1038/s41467-022-28667-0 PMC8956734 35338138

[48] Seldon C, Wong W, Jagsi R, Croke J, Lee A, Puckett L. Remote mentorship in radiation oncology: lessons to share. Adv Radiat Oncol. 2021;6(4):100686. 10.1016/j.adro.2021.100686 PMC8188242 34141954

[49] Holliday EB, Jagsi R, Thomas CR, Wilson LD, Fuller CD. Standing on the shoulders of giants: results from the Radiation Oncology Academic Development and Mentorship Assessment Project (ROADMAP). Int J Radiat Oncol Biol Phys. 2014;88(1):18‑24. 10.1016/j.ijrobp.2013.09.035 PMC4085773 24210670

