# Supplementary material for: Impact of discrimination on training and career of radiation oncologists in France
Source: Clin Transl Radiat Oncol. 2024 Aug 13;48:100840. doi: 10.1016/j.ctro.2024.100840 (PMC11381992; doi:10.1016/j.ctro.2024.100840)
Supplement: Supplementary Data 1 [file mmc1.pdf]

ESMO W4O Survey on the challenges facing oncology professionals in their career development

intro **The European Society for Medical Oncology (ESMO) Women for Oncology (W4O) Committee is running this survey to gather data on the current challenges related to diversity which oncology professionals must deal with at work.**

The survey is anonymous and should take less than 15 minutes to complete.
The ESMO W4O initiative promotes equal access to career-development opportunities.

Thank you for your contribution.

Q1 **Section 1: Demographics**

This section is about who you are

Age:

▼ 21-25 ... >70

Q2 **Gender:**

○ **Female**

○ **Male**

○ **Transgender**

○ **Other, please specify** ____

○ **Prefer not to say**

Q3 **Ethnicity:**

- ◯ **White**
- ◯ **Black**
- ◯ **Asian (East/South East)**
- ◯ **Asian (South)**
- ◯ **Hispanic**
- ◯ **Arab**
- ◯ **Mixed**
- ◯ **Other** ____

Q4 **Country of origin:**

▼ Afghanistan ... Other

Q5 **Are you an ESMO member?**

- ◯ **Yes**
- ◯ **No**

Q6 **Do you live alone?**

- ◯ **Yes**
- ◯ **No**

Q7 **Do you have any children/dependents?**

- ◯ **Yes**
- ◯ **No**

Display This Question: If Do you have any children/dependents? = Yes

Q7a **How many children/dependents do you have?**

Display This Question: If Do you have any children/dependents? = Yes

Q7b **Age:**

Please tick all that apply

- ☐ **Pre-school**
- ☐ **Primary school**
- ☐ **Secondary school**
- ☐ **Adult (living at home)**
- ☐ **Adult (not living at home)**

Display This Question: If Do you have any children/dependents? = Yes

Q8 **Who is the primary child care-taker?**

- ○ **Myself**
- ○ **My spouse/partner**
- ○ **Both**
- ○ **Other family members (e.g. grandparents)**
- ○ **Baby-sitter / nanny**
- ○ **Not applicable**
- ○ **Other (please specify)** ____

Q9 **Section 2: Household duties**

Who does the following duties in your household?

	Always you	Usually you	Both you and your partner	Usually your partner	Always your partner	Another person	Not applicable
**Childcare**	○	○	○	○	○	○	○
**Housekeeping**	○	○	○	○	○	○	○
**Administration**	○	○	○	○	○	○	○
**Grocery Shopping**	○	○	○	○	○	○	○
**Preparing meals**	○	○	○	○	○	○	○
**Laundry**	○	○	○	○	○	○	○
**Other, please specify:**	○	○	○	○	○	○	○

Q10 **Section 3: Place of work**
This section is about your work

What is your country of practice?

▼ Afghanistan ... Other

Q11 **Are you a trainee?**

- ○ **Yes**
- ○ **No**

Q12 **How many years have you been practicing oncology (including years of training)?**

▼ 1 ... 60

Q13 **What is your speciality?**
Please tick all that apply

- ☐ **Medical oncology**
- ☐ **Clinical/Radiation oncology**
- ☐ **Surgical oncology**
- ☐ **Hemato-oncology**
- ☐ **Palliative Care**
- ☐ **Nursing**
- ☐ **Laboratory-based researcher/scientist**
- ☐ **Other, please specify: ____**

Q14 **What is your primary place of work?**

- ○ **Cancer Centre (cancer patients only)**
- ○ **General Hospital (cancer patients and other specialties)**
- ○ **Private outpatient clinic**
- ○ **Pharmaceutical/biotechnology company**
- ○ **Healthcare organisation**
- ○ **Other, please specify: ____**

Q15 **Please specify the % of your working time dedicated to (choices must sum up to 100%):**
Clinical care (%) : _______
Research (%) : _______
Teaching (%) : _______
Management (%) : _______
Administration (%) : _______
Total : ________

Q16 **How many hours per day do you dedicate to work?**

	**Number of hours**
**On working days**	
**On weekends or days off**	

Q17 **How many men and women work in:**
If not applicable please type in 0

	Men	Women
**Your team?**		
**Your department?**		

Q18 **Are you the person heading:**

	Yes	No	Not applicable
**Your team?**	○	○	○
**Your department?**	○	○	○

Display This Question:If Are you the person heading: = Your team? No

18a1 **The person heading your work team is a:**

○ **Man**

○ **Woman**

Display This Question:If Are you the person heading: = Your department? No

18b2 **The person heading your department is a:**

- ◯ **Man**
- ◯ **Woman**

Display This Question:

If Are you the person heading: = Your team? No

And Are you the person heading: =Your department? No

Q19 **Within your job, do you have any managerial or leadership roles?** **<u>Managerial or leadership role:</u> any role/position at workplace that implies high-level responsibilities with the management of a team/group/unit**

- ◯ **Yes**
- ◯ **No**

Display This Question:

If Are you the person heading: = Your team? Yes

Or Are you the person heading: = Your department? Yes

Or Within your job, do you have any managerial or leadership roles? Yes

Q19a **Which of the following positions best describe your role (more than one answer possible)?**

- ☐ **Professor**
- ☐ **Associate Professor**
- ☐ **Dean**
- ☐ **Department Head**
- ☐ **Head of Unit**
- ☐ **Division Head**
- ☐ **Head of Clinical Research Unit**
- ☐ **Head of Clinical Research Division**
- ☐ **Other, please specify:** ____

Q20 **In your country, how do oncology professionals get promoted to higher level of responsibility positions?**
Please tick all that apply

- ☐ **By open job interviews**
- ☐ **By years of experience**
- ☐ **By decisions made by superiors**
- ☐ **Other, please specify:**

____

Q21 **Section 4: Challenges for career progression How important is for you to progress in your career?**

- ○ **Not at all important**
- ○ **Slightly important**
- ○ **Important**
- ○ **Fairly important**
- ○ **Very important**

Q22 **How satisfied are you with your career progression so far?**

- ○ **Not at all satisfied**
- ○ **Slightly satisfied**
- ○ **Satisfied**
- ○ **Fairly satisfied**
- ○ **Very satisfied**

Q23 **Do you think you encountered any obstacles or challenges in your career progression?**

- ○ **Yes**
- ○ **No**

Display This Question: If Do you think you encountered any obstacles or challenges in your career progression? = Yes

Q23a **What obstacles or challenges have you encountered during your career progression? (select up to 3)**

- ☐ **Finding a balance between work and family**
- ☐ **Managing and organizing family commitments**
- ☐ **Lack of mentors/ role models**
- ☐ **Barriers to travel to attend international meetings**
- ☐ **Difficulty to spend time abroad/at a different institute for research fellowship**
- ☐ **Parental leave and difficulties in coming back to work**
- ☐ **Financial constraints**
- ☐ **Social pressure related to cultural gender prejudice about family and domestic responsibilities**
- ☐ **Lack of support from family**
- ☐ **Not being perceived adequate to cover a leadership position**
- ☐ **Lack of support from my manager**
- ☐ **Hostile environment or mobbing**

☐ ⊗ None of the above

☐ Other, please specify: ____________________

Q24 **To what extent have the following aspects <u>been impacted by your career</u>:**

	Not applicable	Not at all	Slightly	Moderately	Very	Extremely
**Friends and social networking**	○	○	○	○	○	○
**Family/Marriage**	○	○	○	○	○	○
**Parental Leave**	○	○	○	○	○	○
**Time dedicated to childcare**	○	○	○	○	○	○
**Leisure activities**	○	○	○	○	○	○

Q25 **To what extent have the following personal choices <u>impacted your career</u>:**

	Not applicable	Not at all	Slightly	Moderately	Very	Extremely
**Difficulty in moving location**	○	○	○	○	○	○
**Reduced working hours**	○	○	○	○	○	○
**Extended parental leave**	○	○	○	○	○	○
**Having children**	○	○	○	○	○	○

Q26 **Section 5: Diversity's impact on career development and barriers for equality**
What level of impact do you think these personal traits have had on your professional career?

	No impact	Minor impact	Moderate impact	Significant impact	Major impact	I don't know
**Ethnicity**	○	○	○	○	○	○
**Sexual orientation**	○	○	○	○	○	○
**Religion**	○	○	○	○	○	○
**Gender**	○	○	○	○	○	○

Q27 **Because of your ethnicity, do you believe that in your professional career you had:**

- ○ **More opportunities**
- ○ **Less opportunities**
- ○ **I think ethnicity didn't affect the opportunities I had in my professional career**

Q28 **Do you think that ethnicity played a role in setting your salary?**

- ○ **Yes**
- ○ **No**
- ○ **I don't know**

Q29 **Do you perceive any pay gap due to ethnicity:**

	Yes	No	I don't know
**at your workplace?**	◯	◯	◯
**in Oncology in your country?**	◯	◯	◯
**in Oncology in general?**	◯	◯	◯

Q30 **Have you experienced bias or discrimination from patients because of your ethnicity?**

◯ **Yes**

◯ **No**

◯ **Prefer not to say**

Q31 **Because of your sexual orientation, do you believe that in your professional career you had:**

◯ **More opportunities**

◯ **Less opportunities**

◯ **I think sexual orientation didn’t affect the opportunities I had in my professional career**

Q32 **Do you think that your sexual orientation played a role in setting your salary?**

◯ **Yes**

◯ **No**

◯ **I don't know**

Q33 **Do you perceive any pay gap due to <u>sexual orientation</u>:**

	Yes	No	I don't know
**at your workplace?**	○	○	○
**in Oncology in your country?**	○	○	○
**in Oncology in general?**	○	○	○

Q34 **Have you experienced bias or discrimination from patients because of your <u>sexual orientation</u>?**

- ○ **Yes**
- ○ **No**
- ○ **Prefer not to say**

Q35 **Because of <u>your religion</u>, do you believe that in your professional career you had:**

- ○ **More opportunities**
- ○ **Less opportunities**
- ○ **I think religion didn’t affect the opportunities I had in my professional career**

Q36 **Do you think that <u>your religion</u> played a role in setting your salary?**

- ○ **Yes**
- ○ **No**
- ○ **I don't know**

Q37 **Do you perceive any pay gap due to religion:**

	Yes	No	I don't know
**at your workplace?**	○	○	○
**in Oncology in your country?**	○	○	○
**in Oncology in general?**	○	○	○

Q38 **Have you experienced bias or discrimination from patients because of your religion?**

- ○ **Yes**
- ○ **No**
- ○ **Prefer not to say**

Q39 **Because of your gender, do you believe that in your professional career you had:**

- ○ **More opportunities**
- ○ **Less opportunities**
- ○ **I think gender didn’t affect the opportunities I had in my professional career**

Q40 **Do you think that gender played a role in setting your salary?**

- ○ **Yes**
- ○ **No**
- ○ **I don't know**

Q41 **Do you perceive gender pay gap:**

	Yes	No	I don't know
at your workplace?	○	○	○
in Oncology in your country?	○	○	○
in Oncology in general?	○	○	○

Q42 **Have you experienced bias or discrimination from patients because of your gender?**

○ **Yes**

○ **No**

○ **Prefer not to say**

Display This Question:If Have you experienced bias or discrimination from patients because of your gender? =Yes

Q42a **Did you receive it from a male of female patient?**

○ **Male**

○ **Female**

○ **Both**

Q43 **Have you experienced bias or discrimination from a senior colleague at work because of your gender?**

○ **Yes**

○ **No**

○ **Prefer not to say**

Display This Question: If Have you experienced bias or discrimination from a senior colleague at work because of your gender? = Yes

Q43a **Did you receive it from a male of female colleague?**

- ○ **Male**
- ○ **Female**
- ○ **Both**

Q44 **Based on your personal knowledge and experience, what do you think are the main barriers that prevent reaching gender parity in the oncology field? (select up to 3)**

- ☐ **Lack of work-life balance**
- ☐ **Lack of role models**
- ☐ **Lack of female professionals' self confidence**
- ☐ **Societal pressures**
- ☐ **Unclear career paths**
- ☐ **Lack leadership development for women**
- ☐ **Lack of paternity leave**
- ☐ **Insufficient maternity leave**
- ☐ **Unconscious bias**
- ☐ **No barriers**
- ☐ ⊗ **None of the above**
- ☐ **Other, please specify:**

____

Q45 **Section 6: Inappropriate behaviour experienced in your professional career**

Have you experienced unwanted sexual comments, attention, advances or any other type of harassment in your workplace?

- ◯ **Yes**
- ◯ **No**
- ◯ **Prefer not to say**

Q46 **Have you witnessed, unwanted sexual comments, attention, advances or any other type of harassment in your workplace?**

- ◯ **Yes**
- ◯ **No**
- ◯ **Prefer not to say**

Q47 **Which of the following options best describe the most inappropriate behaviour you have experienced or witnessed?**
Please select only the most inappropriate behaviour you have experienced or witnessed, even if more than one applies.

- ◯ **Generalised sexist remarks**
- ◯ **Inappropriate sexual advances**
- ◯ **Subtle bribery to engage in sexual behaviours**
- ◯ **Coercive advances to engage in sexual behaviours**

Q48 **Have you reported on harassment that you experienced or witnessed?**

- ◯ **Yes**
- ◯ **No**
- ◯ **Prefer not to say**

Display This Question:If Have you reported on harassment that you experienced or witnessed? = No

Q48a **Why did you decide to not report it?**

- ◯ **Did not think it was important enough**
- ◯ **Did not think anything would be done about it**
- ◯ **Fear of reprisal**
- ◯ **Other, please specify:** ____

Q49 **Section 7: Closing the gender gap**

In order to foster gender equality in the workplace, what approach should be taken in the oncology field? (select up to 3)

- [ ] **Promote work-life balance (e.g. work-life balance policies equally for men and women)**
- [ ] **Seek ways to remove unconscious bias in decision making (e.g. workshop on understanding of unconscious bias)**
- [ ] **Development and leadership training (e.g. mentorship and development programmes)**
- [ ] **Visible leadership commitment towards diversity (e.g. symbolic actions by top management)**
- [ ] **Promote role models (e.g. involving leaders to display possible career paths)**
- [ ] **Promote education on culture on gender equality at work for all workers (men and women)**
- [ ] **Build awareness on the benefits of gender diversity among managers (e.g. workshops on gender diversity)**
- [ ] **Offer and support flexible work (e.g. offering childcare)**
- [ ] **Transparent career paths and salary structures**
- [ ] **Introduce paternity leave**
- [x] **None of the above**
- [ ] **Other, please specify:**

____

Q50 **How much progress do you feel has been made in closing the gender gap in the oncology field compared to when you started working?**

- ◯ **No progress**
- ◯ **Minor progress**
- ◯ **Moderate progress**
- ◯ **Significant progress**
- ◯ **Major progress**
- ◯ **I don't know**

Q51 **Which of the following programmes would you advise ESMO to implement to foster gender equality in oncology? (select up to 3)**

- ☐ **Mentorship programme for female oncologists**
- ☐ **Scholarship to learn from leaders in the field**
- ☐ **Flexible educational / fellowship programmes**
- ☐ **Family-friendly facilities at oncology events**
- ☐ **Online professional career development tools**
- ☐ **Advocating to ease re-entering workplace after parental leave**
- ☐ **Quotas for women in ESMO committees, faculties and events**
- ☐ **Introduce a rule to have gender balance applied to all the aspects of the Society**
- ☐ **Soft-skills training (communication, management, etc)**
- ☐ **Advocating for the introduction of quotas for women in the wider oncology arena**

☐ **Other, please specify:**

____

Q52 **Feel free to share any additional comment you may have:**

____

____

____

____

____

Thank you screen **Thank you for completing the survey!**

Visit ESMO website **for more information on the ESMO W4O Initiative.**
