# Supplementary material for: Impact of discrimination on training and career of radiation oncologists in France
Source: Clin Transl Radiat Oncol. 2024 Aug 13;48:100840. doi: 10.1016/j.ctro.2024.100840 (PMC11381992; doi:10.1016/j.ctro.2024.100840)
Supplement: Supplementary Data 2 [file mmc2.pdf]

1. Données démographiques

Question 1 : Êtes-vous ? *

- Une femme
- Un homme
- Transgenre
- Autre (merci de preciser)
- Ne se prononce pas

Autre (merci de préciser)

Question 2 : Quel âge avez vous ? *

Question 3 : Quel est votre pays de naissance ? *

Question 4 : Êtes vous oncologue-radiothérapeute (ou interne d'oncologie-radiothérapie) ?

- Oui
- Non

Question 5 : Vivez vous seul.e ?

- Oui
- Non

Question 6 : Quel est votre statut marital ?

- Célibataire
- En couple
- Marié.e ou pacsé.e
- Séparé.e ou divorcé.e
- Veuf/Veuve
- Ne se prononce pas

Question 7: Avez-vous des enfants, ou des personnes dépendant de vous ?

- Oui
- Non

Question 7a : Si, oui combien ?

Question 7b: Quel âge ont vos enfants ?

- 0 - 5 ans
- 6 - 11ans
- 10 - 15 ans
- 16- 20 ans
- Plus de 20 ans

(Cochez autant que nécessaire)

Question 8 : Quel est votre mode d'exercice ?

- Interne
- Che.ffe de clinique assistant.e / assistant.e
- Practien.ne hospitalier.e ou en CLCC
- Salarié.e libéral.e
- Associé.e libéral.e
- Attachée.e
- MCU-PH
- PU-PH
- Chef.fe de service
- Autre ? Merci de préciser

Autre ? Merci de préciser

Question 9 : Dans quel type de structure exercez vous ? *

- CHU
- CH, CHG
- Privée
- Centre de lutte contre le cancer
- autres (merci de préciser)

autres (merci de préciser)

2. Pratique médicale

Question 10 : A combien estimez vous vos heures de travail par semaine ?

En semaine (lundi-vendredi)

En week end (samedi-dimanche)

Question 11 : Comment se répartit votre temps de travail ? (en pourcentage)

(Pour un total de 100%)

Au contact du patient

Enseignement

Recherche

Management, gestion

Question 12 : Quel est le nombre d'oncologues-radiothérapeutes dans votre lieu d'exercice ? *

Question 13 : Concernant la répartition des postes d'oncologues radiothérapeutes dans votre lieu d'exercice

Combien d'hommes travaillent dans votre service ?

Combien de femmes travaillent dans votre service ?

Question 14 : Le/la chef.fe de service/comité de direction est ?

- Une femme
- Un homme
- Non concerné.e

Question 15 : Dans votre pratique courante, avez-vous un rôle manégerial (responsablité concernant le management d'une équipe ou d'un groupe) ? *

- Oui
- Non
- Ne se prononce pas

Question 16 : Quelle position correspondrait le plus à votre rôle ?

- Directeur.rice de comité
- Directeur.rice d'unité de recherche
- Chef.fe de service
- Chef.fe de département
- Doyen.ne
- Autre : merci de préciser

Autres : Merci de bien vouloir préciser

Question 17 : Quels sont les aspects de votre profession que vous appréciez le plus ? *

- Relations avec le patient
- Stimulation intellectuelle
- Challenge thérapeutique
- Aspect technique
- Recherche et developpements
- Possibilité de travailler avec d'autres spécialistes
- Prestige de la profession
- Aspect financier
- Répartition du temps de travail

(Cochez autant que nécessaire)

3. Carrière

Question 18 : D'après votre expérience, en général, par quel moyen les oncologues-radiothérapeutes sont-ils promus à des postes à responsabilité ?

- ☐ Par ancienneté
- ☐ Après un entretien d'embauche
- ☐ Par décision de leurs supérieurs
- ☐ Sur concours

Question 19 : Quelle importance donnez vous à la progression de votre carrière ?

- ○ Aucune
- ○ Faible
- ○ Moyenne
- ○ Grande
- ○ Très grande

Question 20: Etes-vous satisfait de votre choix professionnel ? *

- ○ Pas du tout satisfait
- ○ Peu satisfait
- ○ Satisfait
- ○ Plutôt satisfait
- ○ Très satisfait

Question 21 : Pensez-vous avoir rencontré des obstacles dans votre évolution de carrière ? *

- ○ Oui
- ○ Non
- ○ Ne souhaite pas répondre

Question 22 : Quels sont les obstacles parmi ceux listés ci-dessous qui ont eu le plus d'impact sur votre évolution de carrière ?

- [ ] L'équilibre temps de travail/ temps personnel
- [ ] Organisation familiale
- [ ] Manque de mentorat
- [ ] Difficulté d'accès au congrès internationaux
- [ ] Difficulté d'accès à l'exercice ou à des stages à l'étranger
- [ ] Difficulté au retour de congés maternité, paternité, parental
- [ ] Contraintes financières
- [ ] Les discriminations liées au genre
- [ ] Les discriminations liées aux origines socio-ethniques
- [ ] Les discriminations liées à l'orientation sexuelle
- [ ] Manque de soutien familial
- [ ] Manque de soutien des supérieurs
- [ ] Environnement hostile
- [ ] Autres : (merci de préciser)

(Cochez autant que nécessaire)

Question 23 : A quel point votre carrière a t-elle impacté les aspects suivants de votre vie privée ? *

	Non applicable	Pas du tout	Un peu	Modérément	Beaucoup	Fortement
Votre vie sociale (amis...) *	○	○	○	○	○	○
Famille, mariage *	○	○	○	○	○	○
Congé parental *	○	○	○	○	○	○
Temps consacré à vos enfants *	○	○	○	○	○	○
Vos plaisirs, activités personnelles *	○	○	○	○	○	○

Question 24 : A quel point les choix personnels suivants ont impacté négativement votre carrière ? *

	Non applicable	Pas du tout	Un peu	Modérément	Beaucoup	Fortement
Le choix de votre lieu de résidence *	○	○	○	○	○	○
Limiter votre temps de travail *	○	○	○	○	○	○
Prendre un congé parental *	○	○	○	○	○	○
Avoir des enfants *	○	○	○	○	○	○

Question 25 : Pensez-vous que parmi ces critères, certains ont eu une répercussion sur votre carrière ? *

	Non, pas d'impact	Faible impact	Impact modéré	Impact significatif	Impact important	Je ne sais pas
Votre origine ethnique *	○	○	○	○	○	○
Votre origine sociale *	○	○	○	○	○	○
Votre orientation sexuelle *	○	○	○	○	○	○
Votre religion *	○	○	○	○	○	○
Votre genre *	○	○	○	○	○	○

Question 26 : Pensez-vous qu'à cause de votre origine ethnique, vous avez eu ... ?

- ○ Plus d'opportunités de carrière
- ○ Moins d'opportunités de carrière
- ○ Autant d'opportunités de carrière
- ○ Je ne sais pas

Question 27 : Pensez-vous que votre origine ethnique a impacté votre salaire ? *

- ○ Oui
- ○ Non
- ○ Je ne sais pas

Question 28 : Pensez-vous qu'en général l'origine ethnique a une influence sur les inégalités salariales… *

	Oui	Non	Je ne sais pas
Dans votre lieu de travail ? *			
En oncologie-radiothérapie en France? *			
Dans la société en général ? *			

Question 29 : Avez-vous subi des discriminations liées à vos origines ethniques de la part de patients ? *

- Oui
- Non
- Je ne sais pas

Question 30 : Pensez-vous qu'à cause de votre orientation sexuelle, vous avez eu … ?

- Plus d'opportunités de carrière
- Moins d'opportunités de carrière
- Autant d'opportunités de carrière
- Je ne sais pas

Question 31 : Pensez-vous que votre orientation sexuelle a impacté votre salaire ? *

- Oui
- Non
- Je ne sais pas

Question 32 : Pensez-vous qu'en général l'orientation sexuelle a une influence sur les inégalités salariales… *

	Oui	Non	Je ne sais pas
Dans votre lieu de travail ? *			
En oncologie-radiothérapie en France? *			
En oncologie-radiothérapie en général ? *			

Question 33 : Avez-vous subi des discriminations liées à votre orientation sexuelle de la part de patients ? *

- Oui
- Non
- Je ne sais pas

Question 34 : Pensez-vous que votre religion a impacté votre salaire ? *

- Oui
- Non
- Je ne sais pas

Question 35 : Pensez-vous qu'à cause de votre religion vous avez eu ... ?

- Plus d'opportunités de carrière
- Moins d'opportunités de carrière
- Autant d'opportunités de carrière
- Je ne sais pas

Question 36: Pensez-vous qu'en général la religion a une influence sur les inégalités salariales... *

	Oui	Non	Je ne sais pas
Dans votre lieu de travail ? *			
En oncologie-radiothérapie en France? *			
Dans la société en général ? *			

Question 37 : Avez-vous subi des discriminations liées à votre religion de la part de patients ? *

- Oui
- Non
- Je ne sais pas

Question 38 : Pensez-vous que votre genre a impacté votre salaire ? *

- Oui
- Non
- Je ne sais pas

Question 39 : Pensez-vous qu'à cause de votre genre vous avez eu ... ?

- Plus d'opportunités de carrière
- Moins d'opportunités de carrière
- Autant d'opportunités de carrière
- Je ne sais pas

Question 40: Pensez-vous qu'en général le genre a une influence sur les inégalités salariales ... *

	Oui	Non	Je ne sais pas
Dans votre lieu de travail ? *	◯	◯	◯
En oncologie-radiothérapie en France? *	◯	◯	◯
Dans la société en général ? *	◯	◯	◯

Question 41 : Avez-vous subi des discriminations liées à votre genre de la part de patients ? *

- ◯ Oui
- ◯ Non
- ◯ Je ne sais pas

Question 42 : Si oui, de la part d'un patient

- ◯ Homme
- ◯ Femme
- ◯ Les deux

Question 43 : Avez-vous subi des discriminations liées à votre genre de la part d'un supérieur ? *

- ◯ Oui
- ◯ Non
- ◯ Ne se prononce pas

Question 44 : Si oui, de la part d'un.e collègue

- ◯ Homme
- ◯ Femme
- ◯ Les deux

Question 45 : Sur la base de votre expérience personnelle et de vos connaissances, quels sont selon vous les plus grands freins à l'égalité homme-femme en oncologie-radiothérapie ?

- [ ] Déséquilibre de la balance temps personnel/temps professionnel
- [ ] Manque de modèle, mentor
- [ ] Manque de confiance en soi chez les femmes
- [ ] Pression sociale
- [ ] Projets professionnels peu clairs
- [ ] Manque de leadership chez les femmes
- [ ] Manque de congé paternité
- [ ] Congé maternité insuffisant
- [ ] Biais plus inconscient
- [ ] Je ne sais pas
- [ ] Il n'y a pas de barrière
- [ ] Autre : à préciser

Question 46 : Au cours de votre pratique de la médecine ou de votre formation, avez vous été témoin de violences liées à l'identité de l'un.e de vos collègues ?

- [ ] Oui, possiblement liée à l'origine ethnique
- [ ] Oui, possiblement liée à l'origine sociale
- [ ] Oui, possiblement liée à l'orientation sexuelle
- [ ] Oui, possiblement liée au genre
- [ ] Non
- [ ] Je ne sais pas

(Réponses multiples possibles)

Question 47 : Au cours de votre pratique de la médecine ou de votre formation, avez-vous subi un comportement inapproprié, des commentaires sexistes ou une situation de harcèlement sexuel au travail ? *

- ○ Oui
- ○ Non
- ○ Ne se prononce pas

Question 48 : Si oui, quelles ont été les situations inappropriées subies? *

- [ ] Remarques sexistes
- [ ] Avances sexuelles inappropriées
- [ ] Chantage en vue d'obtenir des relations sexuelles
- [ ] Agression sexuelle
- [ ] Autre : à préciser

Question 49 : Au cours de votre pratique de la médecine ou de votre formation, avez vous été témoin de comportements inappropriés, de commentaires sexistes ou d'une situation de harcèlement sexuel au travail ? *

- Oui
- Non
- Ne se prononce pas

Question 50 : Si oui, quelles ont été les situations inappropriées dont vous avez été témoin *

- Remarques sexistes
- Avances sexuelles inappropriées
- Chantage en vue d'obtenir des relations sexuelles
- Agression sexuelle
- Autre : à préciser

Autre : à préciser

Question 51 : Avez-vous déjà rapporté un acte de harcèlement vécu ou dont vous avez témoin ? *

- Oui
- Non
- Ne se prononce pas

Question 52 : Si non, pourquoi avez-vous décidé de ne pas le rapporter ?

- Vous ne trouviez pas cela important
- Vous trouviez cela inutile
- Peur des représailles
- Autre : à preciser

Question 53 :Parmi ces propositions d'amélioration pour soutenir l'égalité et la diversité en oncologie radiothérapie, lesquelles vous semblent les plus adaptées ?

- ☐ Promouvoir une balance temps personnel/temps professionnel équitable entre les hommes et les femmes
- ☐ Travailler sur les biais inconscients et la prise de décision (exemple : workshop)
- ☐ Développer les formations de leadership
- ☐ Mettre en avant des modèles
- ☐ Promouvoir la culture de l'égalité homme-femme au travail
- ☐ Mettre en avant les avantages de la diversité
- ☐ Offrir et permettre des horaires de travail plus flexibles ou des places de garderie
- ☐ Transparence des salaires et des promotions
- ☐ Mettre en avant les congés paternités
- ☐ Aucune des propositions précédentes
- ☐ Autre : à préciser

Question 54 : Comment percevez-vous la diminution des inégalités homme-femme depuis que vous avez commencé à travailler ? *

- ○ Pas de progrès
- ○ Peu de progrès
- ○ Progrès modérés
- ○ Progrès significatifs
- ○ Progrès majeurs
- ○ Je ne sais pas

Question 55 : Parmi ces propositions d'amélioration, lesquelles voudriez-vous mettre en place pour soutenir l'égalité et la diversité en oncologie radiothérapie?

- ☐ Programme de tutorat
- ☐ Financement d'ateliers de prise de responsabilité
- ☐ Programme de formation plus flexible
- ☐ Présence de gardes d'enfant pendant les congrès
- ☐ Outils de développement professionnel en ligne
- ☐ Prime au retour du congé maternité
- ☐ Ajout de quota de femmes dans les institutions, associations, postes clés...
- ☐ Création de programme éducatif spécifique pour le développement en communication, management, leadership
- ☐ Réseau de femmes oncologues radiothérapeutes
- ☐ Ajout de critère de parité dans les sociétés savantes

(Cochez autant que nécessaire)

Question 56 : Avez-vous déjà discuté de sujets liés aux discriminations vécus avec vos collègues ?

- ○ Oui
- ○ Non
- ○ Je ne souhaite pas répondre

Question 57 : Avez-vous déjà eu l'impression d'avoir fait preuve de comportements/remarques discriminatoires envers vos patients ou vos collègues ?

- ☐ Oui, envers vos patients
- ☐ Oui, envers vos collègues
- ☐ Non, jamais
- ☐ Non, je ne pense pas
- ☐ Je ne souhaite pas répondre

4 Vie personnelle

Question 58: Qui garde ou a gardé votre enfant le plus souvent pendant l'enfance ?

- Vous-même
- Votre partenaire
- Les deux à parts égales
- Autres membres de votre famille (grand-parents)
- Nourrice
- Non concerné.e
- Autre : à préciser

Autre : à préciser

Question 59 : Qui a la charge des tâches ménagères suivantes dans votre foyer ?

	Toujours vous	Souvent vous	Vous et votre partenaire	Souvent votre partenaire	Toujours votre partenaire	Une autre personne	Non applicable
Garde d’enfant							
Ménage							
Administratif							
Courses alimentaires							
Préparation des repas							
Linge							

5. Recherche, publications

Question 60 : Avez-vous déjà participé à l'écriture d'un article scientifique ? *

- ○ Oui
- ○ Non

Question 61 : Avez-vous déjà été premier.e auteur.e ? *

- ○ Oui
- ○ Non

Question 62 : Avez-vous déjà été dernièr.e auteur.e ? *

- ○ Oui
- ○ non

Question 63 : Avez-vous déjà présenté votre travail en congrès ?

- ☐ Oui, un poster
- ☐ Oui, une présentation orale
- ☐ Non

Question 64 : Etes-vous membre d'un groupe de recherche ? *

- ○ Oui
- ○ Non

Question 65 : Avez-vous déjà présidé un groupe de travail ? *

- ○ Oui
- ○ Non

Question 66 : Avez-vous déjà été investigateur.e principal.e d'un essai clinique prospectif ? *

- ○ Oui
- ○ Non

Question 67 : Aimeriez-vous l'être ? *

- ○ Oui
- ○ Non
- ○ Je ne sais pas

Question 68 : Avez-vous été ou êtes-vous président.e d'un groupe de recherche collaboratif? *

- Oui
- Non

Question 69 : Si non, avez-vous activement essayé d'avoir ce poste ? *

- Oui
- Non

Question 70 : Avez-vous une thèse de science ? *

- Oui
- Non

Question 71 : Avez-vous un master 1 ? *

- Oui
- Non

Question 72 : Avez-vous un master 2 *

- Oui
- Non

Question 73 : Avez-vous une HDR ? *

- Oui
- Non

Question 74 : Au cours de votre formation avez vous eu un mentor ? *

- Oui
- Non

Question 75 : Si oui, avez-vous eu des difficultés à trouver un mentor ? *

- Oui
- Non

Question 76 : Si oui, votre mentor était-il.elle ? *

- Une femme
- Un homme

Commentaires libres :
